# Chronic endometritis increases the recurrence of endometrial polyps in premenopausal women after hysteroscopic polypectomy

**DOI:** 10.1186/s12905-023-02232-3

**Published:** 2023-02-25

**Authors:** Dacheng Qu, Yue Liu, Honggui Zhou, Zhibiao Wang

**Affiliations:** 1 State Key Laboratory of Ultrasound in Medicine and Engineering, College of Biomedical Engineering, Chongqing Medical University, Chongqing, 400016 China; 2 Chongqing Key Laboratory of Biomedical Engineering, Chongqing Medical University, Chongqing, 400016 China; 3 Department of Obstetrics and Gynecology, Non-Invasive and Micro-Invasive Laboratory of Gynecology, Affiliated Hospital of North Sichuan Medical College, Nanchong, 637000 China; 4 Non-Invasive and Micro-Invasive Laboratory of Gynecology, Affiliated Hospital of North Sichuan Medical College, Nanchong, 637000 China

**Keywords:** Recurrence, Chronic endometritis, Endometrial polyps, Hysteroscopic polypectomy, Risk factor

## Abstract

**Background:**

The reported recurrence rate of endometrial polyps (EPs) after hysteroscopic polypectomy varied widely, and the factors influencing the recurrence of EPs are still controversial. Furthermore, the known definite independent risk factors are almost unchangeable, such as the number of EPs and previous polypectomy history. The aim of this study was to evaluate the impact of chronic endometritis (CE) on the recurrence of EPs in premenopausal women who underwent hysteroscopic polypectomy.

**Methods:**

A retrospective study was conducted at a university-affiliated hospital. Premenopausal women who underwent hysteroscopic polypectomy were enrolled, and those with definite confounding factors for polyp recurrence were excluded, including endometriosis and previous polypectomy history. A total of 233 women were enrolled in this study, including 64 (27.5%) cases with CE and 169 (72.5%) cases without CE. CE was diagnosed via immunohistochemical detection of CD138 on the endometrial specimen. Comparison of the recurrence rate of EPs was performed in women with or without CE at each monitoring stage (i.e., at 3, 6, 9 and 12 months) after hysteroscopic polypectomy.

**Results:**

The recurrence rates of EPs at one year in patients with and without CE were 26.6% (95% confidence interval [CI] 15.8–37.4%) and 9.5% (95% CI 5.0–14.0%), respectively, with an overall recurrence rate of 14.2% (95% CI 9.7–18.7%). The hazard ratio (HR) for EPs recurrence in the EPs with CE cohort versus the EPs without CE cohort was 3.08 (95% CI 1.56–6.09) (*P* = 0.001). Similarly, the recurrence rate of EPs was significantly higher in women with CE than in those without CE at each monitoring stage (i.e., 3, 6 and 9 months). CE and multiple EPs were risk factors for EPs recurrence. The HR for EPs recurrence in the EPs with CE cohort compared with the EPs without CE cohort was 3.06, after adjustment for the number of EPs.

**Conclusions:**

CE was a harmful factor for the recurrence of EPs in premenopausal women after hysteroscopic polypectomy. Thus, routine screening for CE during hysteroscopic polypectomy was needed. Frequent monitoring was needed for multiple EPs as the number of EPs also contributed to polyp recurrence.

## Background

Endometrial polyps (EPs) represent a common benign gynecological condition that manifests via excessive local hyperplasia of the interstitial and endometrial glands. The prevalence of EPs ranges from 7.8 to 34.9% across different populations [1]. Although the pathogenesis and exact cause of EPs were unknown, chronic inflammation in the endometrium may result in the formation of EPs [2]. The hyaline thickening of vessels was the common morphological vascular alteration of chronic endometritis (CE), which was very similar to the thick-walled vessels of EPs [3]. The increased expression of transforming growth factor-beta 1 (TGF-β1), vascular endothelial growth factor (VEGF), tumor protein P73 (TP73), tumor protein P63 (TP63) and BAX (BCL-2 associated X protein) transcript variant alpha demonstrated the dominance of proliferative and anti-apoptotic activity in CE. This may potentially promote the development and recurrence of EPs [4].

CE involves persistent local inflammation of endometrium, characterized by the presence of plasma cells infiltrating the interstitium [5, 6]. CE is usually asymptomatic or presents only with mild symptoms, such as abnormal uterine bleeding (AUB), pelvic pain, dyspareunia and leucorrhea, as well as infertility and miscarriage, all of which are very similar to the symptoms of EPs [7]. Immunohistochemistry for the plasmacyte marker CD138 is currently the most reliable diagnostic method for CE, which is superior to haematoxylin and eosin (HE) staining and hysteroscopic diagnosis [8–11]. However, the spontaneous cure rate of CE reported in a randomized clinical trial was as low as only 12.7% [12]. The optimal treatment currently for CE is oral antibiotics [13, 14].

EPs are associated with AUB, infertility and recurrent reproductive failure [1, 2, 15]. Hysteroscopic polypectomy is considered as the gold standard treatment for EPs [15, 16]. Many studies have reported that the recurrence rate of EPs at 12 months after hysteroscopic polypectomy ranged widely from 5.6 to 31.4% [17–19]. A large number of EPs, endometriosis and previous polypectomy history are found to be independent risk factors for the recurrence of EPs [18, 19]. In addition, late menopause and obesity increase the occurrence of EPs [20]. However, the factors influencing the recurrence potential of EPs after hysteroscopic polypectomy remain rather controversial. Furthermore, the known definite independent risk factors are almost unchangeable, such as the number of EPs and previous polypectomy history, and thus, concealed changeable factors need to be explored. CE was involved in the formation of EPs. Was CE also involved in the recurrence of EPs after hysteroscopic polypectomy?

In the retrospective study, we investigated whether CE increased the recurrence of EPs in premenopausal women after hysteroscopic polypectomy.

## Methods

### Study design

Between January 2019 and January 2020, a retrospective study was conducted at the Affiliated Hospital of North Sichuan Medical College (China) for women who underwent hysteroscopic polypectomy. 35 patients without regular surveillance in 12 months were excluded. Finally, a total of 233 premenopausal women after hysteroscopic polypectomy were enrolled in this study, including 64 (27.5%) cases with CE and 169 (72.5%) cases without CE. The expression of CD138 in the endometrium was analyzed by immunohistochemistry to identify CE. Comparison of the recurrence rate of EPs was performed in women with and without CE at each monitoring stage (i.e., at 3, 6, 9 and 12 months).

Patients were eligible for inclusion if they had enough endometrial specimen for immunohistochemical analysis of CD138 to identify CE, were premenopausal women (18–50 years) and had no severe systemic diseases. The exclusion criteria were pelvic inflammatory disease (PID) occurring in the 12 months after hysteroscopic polypectomy, previous endometrial polypectomy history, endometriosis, endometrial hyperplasia, and use of hormone therapy or antibiotics. This study was conducted in accordance with the principles of the Declaration of Helsinki.

### Hysteroscopic resection and endometrial biopsy

Hysteroscopic polypectomy was conducted in the follicular phase with a bipolar plasmakinetic resection system under intravenous anesthesia using a 3-mm 15° inside rigid hysteroscope and an 8.5-mm outside sheath (Olympus, Tokyo, Japan). All operations were performed by the same physician (DC Qu). Endometrial samples were obtained visually using an unpowered plasma cutting ring from the upper uterine cavity, while hysteroscopic polypectomy was performed to remove local polyps. Samples of polyps and endometrium were detected for routine histologic analysis after hysteroscopic polypectomy, not only to confirm the diagnosis of EPs, but also to show whether there was malignant transformation or endometrial lesion.

### Immunohistochemistry

The diagnosis of CE should be made first by looking to the morphology of the field and the presence of plasma cell with or without lymphoid follicles. If the morphology is controversial, immunohistochemistry for the plasmacyte marker CD138 was needed. A histopathology consultant performed immunohistochemical detection of CD138 on the endometrial specimen slices as previously described [11, 21]. Five-micrometer sections were cut from the paraffin blocks. Deparaffinization, antigen retrieval, antibody staining, hematoxylin counterstain and 3,3′-diaminobenzidine chromogen (DAB) color development were fully automated in a Leica BondMax autostainer (Leica BioSystems). The clone of anti-CD138 monoclonal antibody used in our study was MI15 Cell Marque (Fuzhou Maixin Biotechnology Co., Ltd., Fuzhou, China). For the diagnosis of CE, CD138 positive cells was counted in endometrial stroma under a light microscope (400 × magnification) (Olympus, Tokyo, Japan). CD138 expression was judged as positive if yellow or brown staining of specific cell membrane/cytoplasm was seen. Although currently there is no specific guidelines about the required number of plasma cells required for the diagnosis, CE was diagnosed by at least 1 CD138 positive cell per 10 high-power fields in this study, as widely used in other studies. At least 50 high-power fields were examined for each specimen [11, 21].

### Follow up

Women who underwent hysteroscopic polypectomy at our hysteroscopic center were surveilled routinely. Three dimensional colour-flow doppler transvaginal ultrasonography was performed to evaluate the uterine cavity condition at 3, 6, 9 and 12 months after hysteroscopic polypectomy. All ultrasound examinations were performed with a Voluson E8 ultrasound system and a 5 to 9 MHz endocavitary transducer (GE Healthcare, Kretz, Zipf, Austria).

### Statistical analyses

For all statistical analyses, we used SPSS version 22.0 (SPSS, Inc, Chicago, IL, USA), and a *P* value < 0.05 was considered to be statistically significant. After analyzing the distribution of the data and confirming that the age and body mass index (BMI) of the population were not normally distributed, we adopted the Mann–Whitney *U* test to analyze age and BMI expressed as median (interquartile range). Categoric variables were compared using the chi-square test. The chi-square test was used to compare the recurrence of EPs between each group at each stage. The Kaplan–Meier method with the log-rank test was used to estimate recurrence-free survival and to compare recurrence rates between the two groups. Univariate and multivariate Cox regression models were used to assess the factors associated with the recurrence of EPs. Factors for which *P* < 0.2 on univariate analyses were included in the subsequent multivariable model.

## Results

### Comparison of general and clinical features of EPs patients with and without CE

The prevalence of CE in the population was 27.5% (64/233). The demographic details and clinical features of the two groups of patients were shown in Table 1. No statistically significant differences in demographic details and clinical features were found between the two groups of patients (all *P* > 0.05).

**Table 1:** Baseline demographics of the enrolled patients

Characteristics	EPs with CE (n = 64)	EPs without CE (n = 169)	*P* value
Age, year (median (Q3–Q1))	33 (9)	34 (8)	0.092
BMI, kg/m^2^ (median (Q3–Q1))	21.7 (3.9)	22.4 (4.0)	0.120
Polyp number, n (%)			0.713
Solitary	17 (26.6%)	49 (29.0%)	
Multiple	47 (73.4%)	120 (71.0%)	
Polyp size (cm), n (%)			0.314
≤ 1	12 (18.8%)	20 (11.8%)	
> 1, ≤ 2	49 (76.6%)	136 (80.5%)	
> 2	3 (4.7%)	13 (7.7%)	

*EPs* endometrial polyps, *CE* chronic endometritis, *BMI* body mass index

### Comparison of EPs recurrence rates between patients with and without CE

**Fig. 1:**
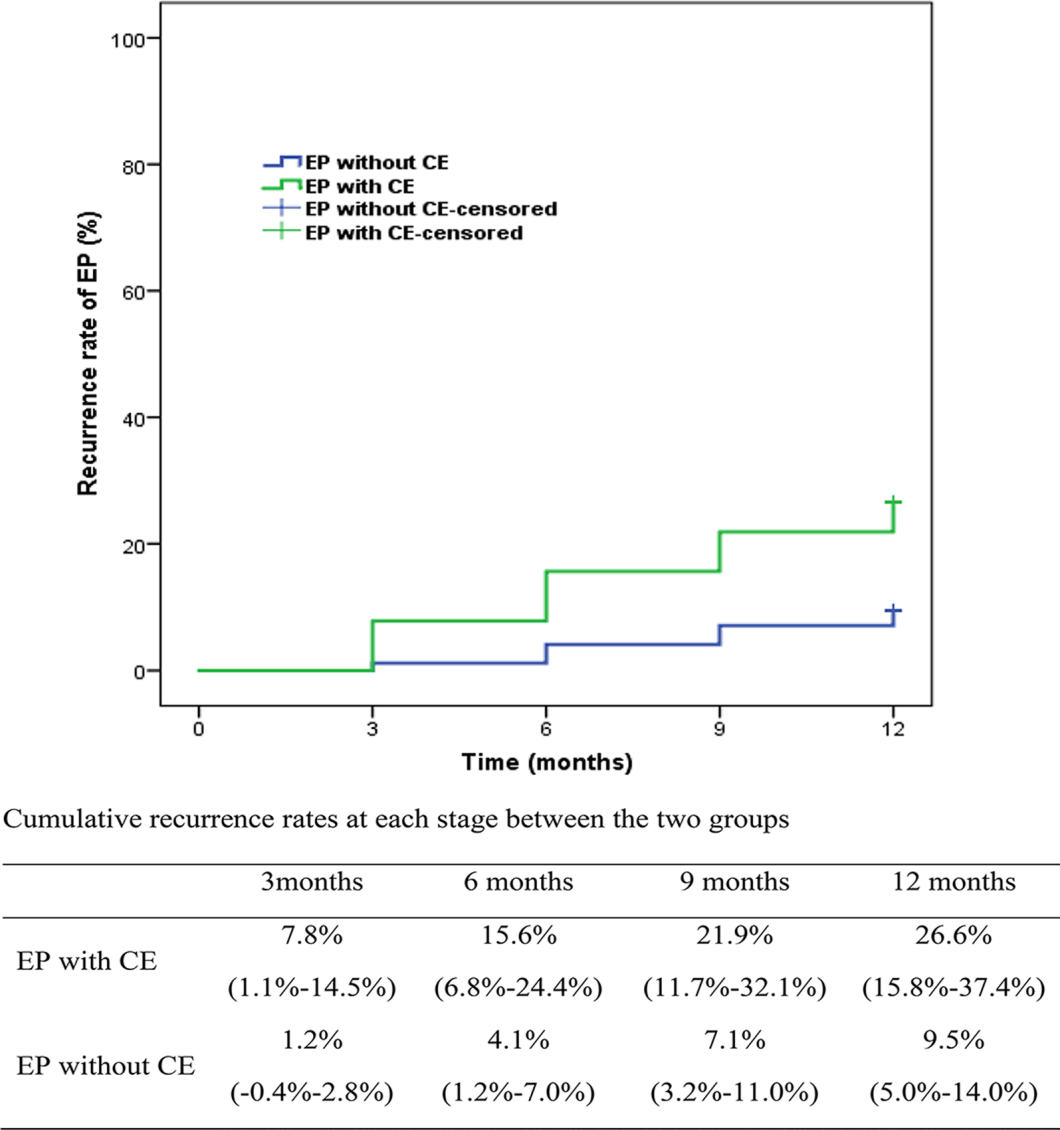
Comparison of EPs recurrence rates between the patient groups with and without CE at each monitoring stage over the 12-month follow-up (all *P* < .05 at 3, 6, 9 and 12 months). Values are rate (95% confidence interval). EPs—endometrial polyps; CE—chronic endometritis

During the 1-year follow-up, a total of 33 women experienced EPs recurrence, including 17 cases with CE and 16 cases without CE. The recurrence rates of EPs with CE and without CE were 26.6% (95% confidence interval [CI] 15.8–37.4%) and 9.5% (95% CI 5.0–14.0%), respectively, with an overall cure rate of 14.2% (95% CI 9.7–18.7%) (Fig. 1). The recurrence rate of EPs was higher in patients with CE (*P* = 0.001). The HR for EPs recurrence in the EPs with CE cohort versus the EPs without CE cohort was 3.08 (95% CI 1.56–6.09, *P* < 0.01; Table 2).

**Table 2:** Risk factors for recurrence of EPs from univariate and multivariate Cox regression analyses

Characteristics	HR (95% CI)	*P* value
Univariate analysis		
Age	1.00 (0.95–1.06)	0.996
BMI	0.93 (0.81–1.05)	0.245
Polyp size (cm) (vs. ≤ 1)		
> 1, ≤ 2	1.54 (0.16–14.80)	0.709
> 2	2.57 (0.35–18.89)	0.353
Multiple EPs versus solitary EP	3.02 (1.06–8.60)	0.038
CE	3.08 (1.56–6.09)	0.001
Multivariate analysis		
CE	3.06 (1.54–6.05)	0.001
Multiple EPs versus solitary EP	2.99 (1.05–8.91)	0.040

*BMI* body mass index, *EPs* endometrial polyps, *EP* endometrial polyp, *CE* chronic endometritis

Similarly, in the group with CE, there were 5 cases of EPs recurrence at 3 months, 10 cases at 6 months, and 14 cases at 9 months. In the group without CE, there were 2 cases of EPs recurrence at 3 months, 7 cases at 6 months, and 12 cases at 9 months. The recurrence rates of EPs were significantly higher in women with CE than in those without CE at each monitoring stage, i.e., 7.8% (95% CI 1.1–14.5%) versus 1.2% (95% CI − 0.4 to 2.8%, *P* = 0.018) at 3 months, 15.6% (95% CI 6.8–24.4%) versus 4.1% (95% CI 1.2–7.0%, *P* = 0.003) at 6 months, and 21.9% (95% CI 11.7–32.1%) versus 7.1% (95% CI 3.2–11.0%, *P* = 0.001) at 9 months (Fig. 1).

### Factors associated with the recurrence of EPs

Univariate and multivariate Cox regression models were used to assess the factors associated with the recurrence of EPs. Univariate analysis showed that the recurrence of EPs was not affected by age, BMI or polyp size, but significantly higher EPs recurrence rates were observed in women with multiple EPs and with CE compared with the rates in patients without the respective conditions (Table 2, *P* < 0.05 and *P* < 0.05, respectively). Multivariate analysis confirmed that CE was a harmful factor for the recurrence of EPs (HR 3.06, 95% CI 1.54–6.05, *P* = 0.001). In addition, as previously mentioned [18, 19], the presence of multiple EPs also contributed to the recurrence of EPs (HR 2.99, 95% CI 1.05–8.91, *P* = 0.040).

## Discussion

In this retrospective cohort study of 233 participants who underwent hysteroscopic polypectomy as treatment for EPs, the recurrence rates of EPs were significantly higher in women with CE than in those without CE at each monitoring stage, i.e., at 3, 6, 9 and 12 months. These findings indicated a harmful role of CE in the recurrence of EPs in premenopausal women after hysteroscopic polypectomy. The HR for EPs recurrence in the EPs with CE cohort compared with the EPs without CE cohort was 3.06, after adjustment for the number of EPs.

Concealed changeable factors for the recurrence of EPs need to be explored, as the known independent risk factors are almost unchangeable, such as the number of EPs and previous polypectomy history [18]. In this study, the overall recurrence rate of EPs in premenopausal women was 14.2% at 12 months after hysteroscopic polypectomy, which is consistent with rates reported in previous studies [17, 22–24]. Although, after subgroup analysis according to CE, the recurrence rate of EPs in premenopausal women increased to 26.6%, and CE played a harmful role in the recurrence of EPs. To the best of our knowledge, our study is the first to report the effect of CE. Similarly, the recurrence rate of EPs was significantly higher in women with CE than in those without CE at each monitoring stage, i.e., 7.8% versus 1.2% at 3 months, 15.6% versus 4.1% at 6 months, and 21.9% versus 7.1% at 9 months. This may be a possible explanation for the wide range of the EPs recurrence rates (5.6–31.4%) during 1-year follow-up reported before [17–19]. CE may have been a concealed factor for the recurrence of EPs. The underlying mechanism of the harmful effects of CE is currently unknown. One possible explanation is that the vessel axis of EPs may actually originate from the evolution of vascular changes associated with endometritis [3]. Secondly, the dominance of proliferative and anti-apoptotic activity in CE may result in the formation and recurrence of EPs [4]. TGF-β1, VEGF, TP73 and TP63 were overexpressed in CE. Finally, compared with non-polypoid endometrium, endometrium of EPs and CE had a lower density of NK cells [25]. This may potentially lead to in situ growth and recurrence of EPs after hysteroscopic polypectomy.

CE is a disease involving destruction of the homeostasis between microorganisms in the endometrium and the host immune system. The most common bacterial pathogens causing CE are reported to be *Enterobacteriaceae*, *Enterococcus*, *Streptococcus*, *Staphylococcus*, *Mycoplasma*, and *Ureaplasma* [26]. Similarly, another study found that the most abundant genus in the microbiota of endometrium with CE is non-*Lactobacillus* [27]. Antibiotics are effective for treating CE [8, 21, 28–30], as it is caused by bacterial pathogens. Although CE was an independent risk factor for polyp recurrence, unlike unchangeable factors, CE can be changed easily by treatment of antibiotics. The one course cure rate of CE was 72.6% treated with oral doxycycline 200 mg daily for 14 days and 89.8% treated with oral levofloxacin 500 mg and tinidazole 1000 mg daily for 14 days [8, 21].

In the current study, to better evaluate the impact of CE on polyp recurrence, patients with definite external confounding factors for polyp recurrence, including endometriosis and previous polypectomy history, were excluded. Univariate analysis was used to determine the relationship between the recurrence of EPs and its potential risk factors, including age, BMI, polyp size, number of EPs and CE. According to the previous studies [17, 18], polycystic ovary syndrome, infertility, gravidity and parity were not included for analysis. Multiple EPs and CE were associated with the potential recurrence of EPs (*P* < 0.05). The HR for EPs recurrence in the multiple EPs cohort compared with the solitary EP cohort was 3.02, which is consistent with previous studies, showing a 3.45 HR with a high number of EPs [18]. After adjusting for CE, The HR of EPs number was 2.99, which may truly reflect the impact of the EPs number. Thus, frequent monitoring was needed for multiple polyps after hysteroscopic polypectomy. Simultaneously, the recurrence of EPs was not affected by age, BMI, or polyp size, which are concurrent with previous studies [18, 19].

There are some limitations in this study. For example, because this was a retrospective study, we could not take into account enough factors affecting the recurrence of EPs, and other concealed factors need to be explored. Furthermore, CE treated with antibiotics cohort was not included in our study, we do not know whether the effect of CE on the recurrence of EPs can be eliminated when treated with antibiotics.

## Conclusions

In conclusion, this study demonstrated that CE was a harmful factor for the recurrence of EPs in premenopausal women after hysteroscopic polypectomy. Thus, routine screening for CE during hysteroscopic polypectomy was needed. Frequent monitoring was needed for multiple EPs as the number of EPs also contributed to polyp recurrence. The role of CE in the recurrence of EPs and whether cured CE can provide prevention to eliminate the harmful effect need to be further confirmed by prospective trials with larger sample sizes.

## Data Availability

The datasets used and/or analysed during the current study are available from the corresponding author on reasonable request.

## Abbreviations

EPs: Endometrial polyps
CE: Chronic endometritis
